# Successful use of dupilumab in recalcitrant pediatric atopic dermatitis-like graft-versus-host disease: A case series

**DOI:** 10.1016/j.jdcr.2023.11.003

**Published:** 2023-11-14

**Authors:** Lina Belmesk, Afshin Hatami, Julie Powell, Victor Kokta, Jerome Coulombe

**Affiliations:** a Division of Dermatology, Department of Medicine, Universite de Montreal, Montreal, Quebec, Canada; b Division of Dermatology, Department of Pediatrics, CHU Sainte-Justine, Universite de Montreal, Montreal, Quebec, Canada; c Division of Pathology, Department of Pediatrics, CHU Sainte-Justine, Universite de Montreal, Montreal, Quebec, Canada

**Keywords:** atopic dermatitis, dupilumab, GvHD, immunology, Th2

## Introduction

Graft-versus-host disease (GvHD) is one of the most severe complications of allogenic hematopoietic stem cell transplantation (HSCT). Although GvHD often presents as a multiorgan disease, the first and most common clinical manifestations are cutaneous. While the number of indications for allogenic HSCT is expanding, dermatologists are challenged with various forms of cutaneous GvHD. Atopic dermatitis-like (AD-like) GvHD has been increasingly recognized by dermatologists over the last decade.

Tanasescu et al^1^ first reported a new subtype of cutaneous GvHD in a patient with eczema-like GvHD in 1999. In 2013, Wei et al subsequently reported the largest case series describing 11 patients with AD-like GvHD following allogenic HSCT.^2^ The main clinical features observed were pruritus, xerosis, dermatitis, scaling, and perifollicular accentuation. Peripheral eosinophilia and increased immunoglobulin E (IgE) levels have also been described.^2^ Histologic findings are comparable to AD histopathology, and include spongiosis, acanthosis, and lymphocytic infiltrates.^2^ Marker of classic GvHD: basal layer vacuolization, scattered eosinophils, and keratinocytes necrosis, were also noted in a majority of patients.^2^ AD-like GvHD often follows a chronic course and requires both systemic immunosuppressive therapy and topical treatments to improve patient’s quality of life.

Systemic corticosteroids remain the cornerstone and first-line treatment for moderate to severe cutaneous GvHD alone or in combination with other immunosuppressive therapies. Second-line therapies include mycophenolate mofetil (MMF), methotrexate, systemic calcineurin inhibitors, rituximab, and ruxolitinib.^3^^,^^4^ Other combined treatment modalities for cutaneous GvHD are topical corticosteroids and calcineurin inhibitors (TCI), phototherapy (nbUVB), antipruritic agents, and extracorporeal photopheresis.^3^^,^^4^

Larijani et al^5^ recently reported 4 pediatric patients with AD-like GvHD, with AD findings on histopathology, successfully treated with dupilumab a monoclonal antibodies targeting interleukin (IL)-4Rα. We report a series of 4 pediatric patients with AD-like GvHD, following allogenic HSCT, recalcitrant to conventional therapies and successfully treated with dupilumab. All 4 patients achieved almost complete skin clearance with marked reduction in pruritus and rapid tapering of their other immunosuppressive therapies. None of our patient had signs of hepatic or gastrointestinal GvHD. This is the first case series including toddlers with AD-like GvHD who have been treated with dupilumab.

## Case report

### Case 1

**Fig 1:**
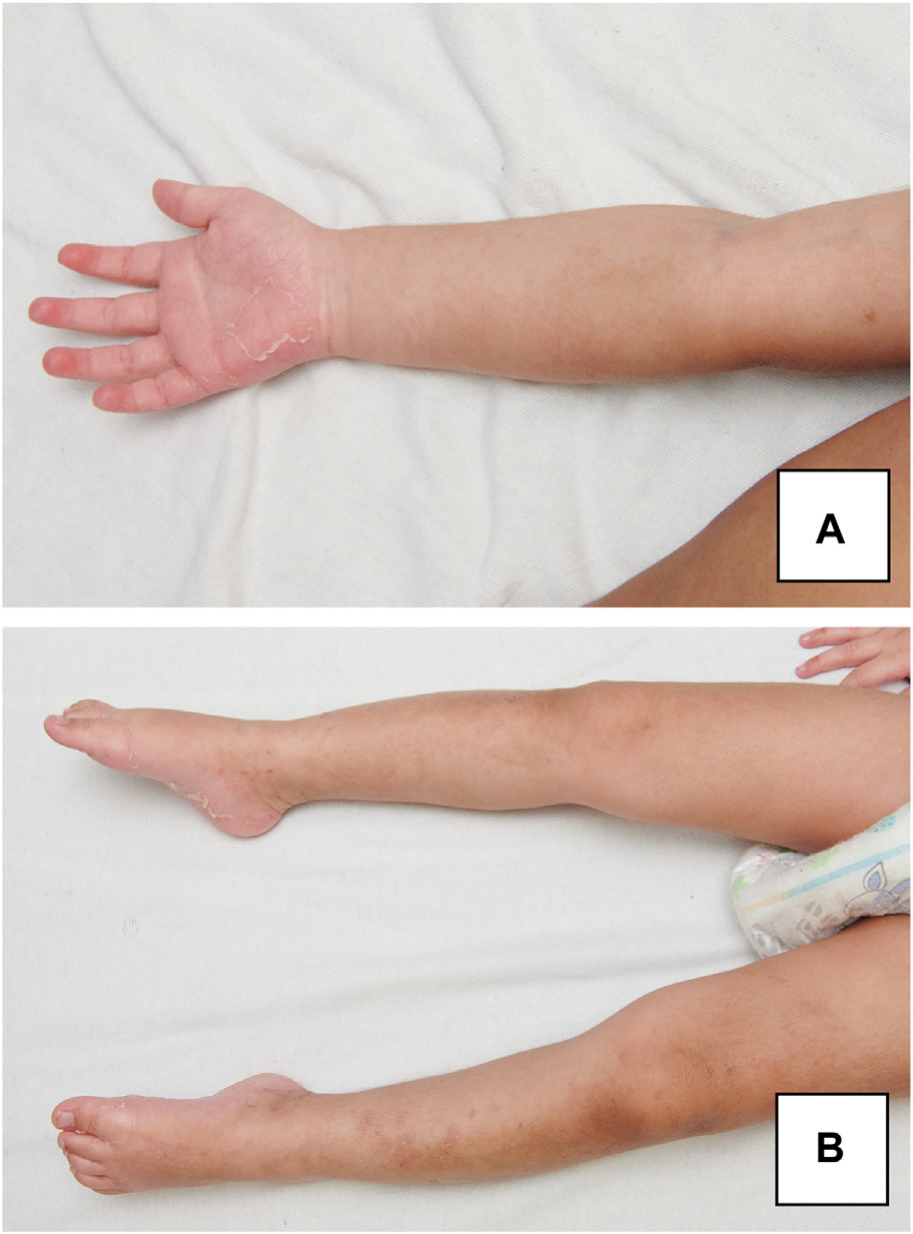
AD-like GvHD in an 3-year-old boy who presented with large ill-defined excoriated eczematous plaques on his *lower* limbs and eventually trunk. He also developed desquamation of his palms (**A**) and soles (**B**) evolving in a widespread distribution commonly seen in classic acute GvHD. *AD-like*, Atopic dermatitis-like; *GvHD*, graft-versus-host disease.

**Fig 2:**
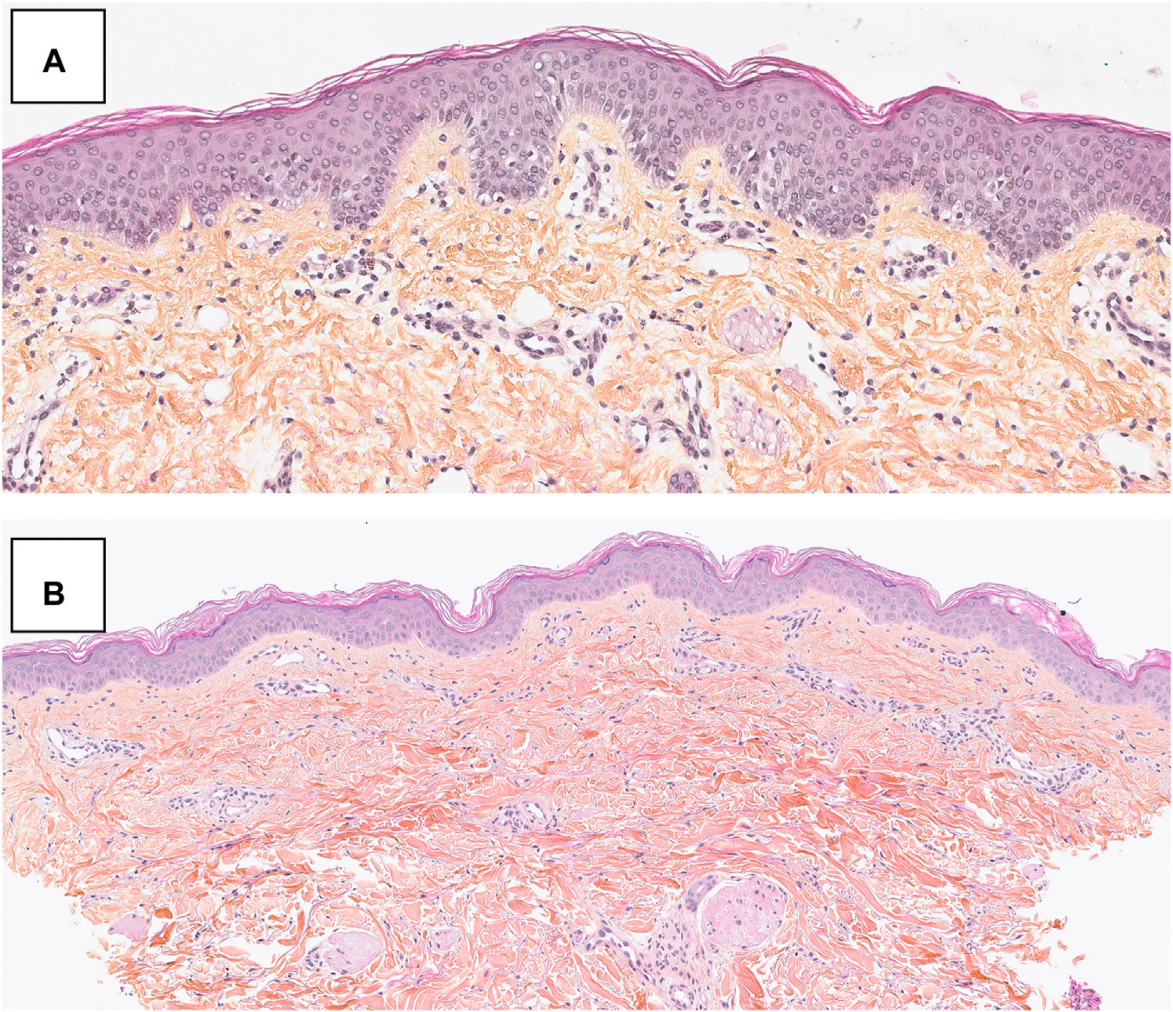
**A,** x350 HES; Case 1 histology showed a mild acute superficial perivascular spongiotic dermatitis with mild lymphohistiocytic infiltrates and secondary pigmentary incontinence compatible with an eczematous dermatitis. **B,** x200 HES; Case 3 histology reveled a moderate acute and subacute spongiotic dermatitis, perivascular mild lymphohistiocytic infiltrate, absence of eosinophils, neutrophils, and plasma cells. A diagnosis of AD-like GvHD was made in both cases. *AD-like*, Atopic dermatitis-like; *GvHD*, graft-versus-host disease; *HES*, hematoxylin and eosin.

A 3-years-old boy born with a severe combined immunodeficiency secondary to a deficiency in purine nucleoside phosphorylase, underwent allogenic HSCT from a human leukocyte antigen-identical unrelated donor. He had no personal or familial history of atopy (Table I). Donor history for atopy was unknown. He initially developed a debilitating generalized pruritus without primary lesions causing nocturnal awakenings. On day 145, he developed large ill-defined widespread erythemato-squamous plaques on his trunk and lower limbs. He also had desquamation of his palms and soles evolving in a widespread distribution, a common sign seen in cutaneous GvHD (Fig 1). A diagnosis of AD-like GvHD was made. Histology was consistent with an eczematous dermatitis and showed an acute to subacute superficial perivascular spongiotic dermatitis with mild lymphohistiocytic infiltrates and secondary pigmentary incontinence (Fig 2, *A*). After failure of several systemic immunosuppressive therapies, including prednisone, rituximab, MMF, sirolimus, and ruxolitinib, as well as high-potency topical corticosteroids and TCI, he was initiated on dupilumab. Three days after his first dose, pruritus was significantly reduced, and after a week, he had complete skin clearance and was discharged from the hospital. After 4 months, he has successfully been tapered off prednisone, MMF, and ruxolitinib and now only requires a low dose of sirolimus post-HSCT and dupilumab 300 mg subcutaneous (SC) each month.

**Table I:** Patients’ demographics and clinical characteristics

Case	Age y	Sex	Primary disease	Conditioning regimen	GvHD prophylaxis	History of atopy	Systemic therapies	Topical CS	Other topical treatments	Photo therapy	Response
1	3	M	SCID PNP	Busulfan, Fludarabine Alemtuzumab	CsA, MMF	No	Prednisone CsA MMF Sirolimus	+	+	+	GvHD resolved Remains on dupilumab and low doses of sirolimus
2	8	M	Fanconi anemia	Fludarabine CyC	ATG, CsA, MMF	No	Prednisone Sirolimus Gabapentin	+	+	+	Mild GvHD Remains on dupilumab
3	2	M	Wiskott-Aldrich syndrome	Busulfan, Fludarabine Alemtuzumab	CsA, MMF	Yes	Prednisone CsA MMF Tacrolimus Sirolimus Ruxolitinib Rituximab	+	+	+	GvHD resolved Complete clearance Off dupilumab and all other systemic therapies
4	5	F	Medullary aplasia	Fludarabine Alemtuzumab	CsA	No	CsA	+	**+**	-	Mild GvHD, no itch CsA weaning off remains on dupilumab

*ATG*, Anti-thymocyte globuline; *CsA*, cyclosporine A; *CyC*, cyclophosphamide; *GvHD*, graft-versus-host disease; *MMF*, mycophenolate mofetil; *PNP*, purine nucleoside phosphorylase; *SCID*, severe combined immunodeficiency.

### Case 2

**Fig 3:**
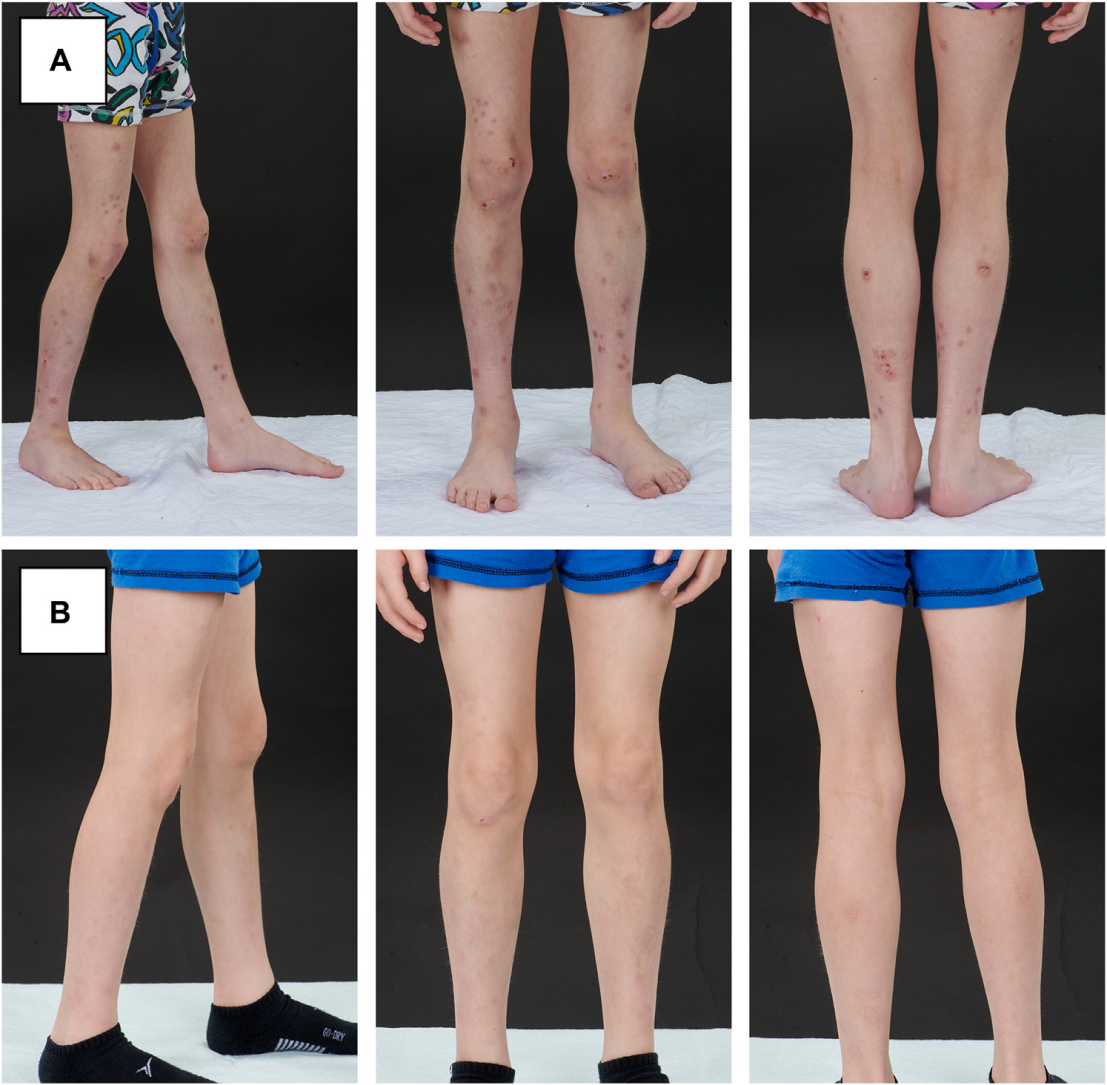
AD-like GvHD in an 8-year-old boy who presented with multiple papules, nodules, and lichenified plaques (**A**). Significant improvement was observed after 8 weeks of dupilumab treatment, with complete resolution of the lesions (**B**). As a result, the patient was able to discontinue his other immunosuppressants used for treating his cutaneous GvHD. *AD-like*, Atopic dermatitis-like; *GvHD*, graft-versus-host disease.

An 8-years-old boy diagnosed with severe Fanconi anemia underwent allogenic HSCT and additional cord blood stem cell transplantation. He was known for a VACTERL syndrome and epilepsy but had no personal or familial history of atopy (Table I). The donor was a human leukocyte antigen-identical sibling. On day 188, he developed multiple inflammatory follicular papules with a prurigo nodular-like appearance on the lower limbs. Biopsy was not performed. Patient developed a refractory and pronounced pruritus and was started on dupilumab 200 mg SC each 2-weeks after failure of systemic prednisone, high potency topical corticosteroids, nbUVB, antihistamines, and gabapentin. After 1 month on dupilumab, he noted a marked reduction in the number of his skin lesions, a marked decrease in his Eczema Area Severity Index (EASI) score and resolution of his pruritus (Table II). The patient achieved almost complete clearance after 8 weeks of dupilumab and was able to quickly decrease the dose of his other immunosuppressive therapies (Fig 3).

**Table II:** Patients’ dermatologic severity scores before and after dupilumab

Case	Dupilumab	BSA (%)	EASI Score	IGA Score	DLQI Score
1	Predupilumab	48	31.5	4	26
	Postdupilumab	0	1.2	1	1
2	Predupilumab	10	17	4	19
	Postdupilumab	1	2.6	0	9
3	Predupilumab	55	38	4	25
	Postdupilumab	0	0	0	0
4	Predupilumab	26	10.8	3	16
	Postdupilumab	6	3	1	3

*BSA*, Body surface area; *DLQI*, Dermatology Life Quality Index; *EASI*, Eczema Area Severity Index; *IGA*, Investigator Global Assessment Scale.

### Case 3

A 2-year-old boy known for Wiskott-Aldrich syndrome with immune deficiency, thrombocytopenia, and mild atopic dermatitis initially controlled with mild potency topical corticosteroids and TCI underwent allogenic HSCT (Table I). On day 31, due to prednisone tapering, he developed multiple severe erythemato-squamous ill-defined plaques on his limbs and buttock. Histology showed acute spongiotic dermatitis with perivascular mild lymphohistiocytic infiltrate (Fig 2, *B*). He failed topical treatments, prednisone, ruxolitinib, and nbUVB. A diagnosis of AD-like GvHD was made. He achieved complete clearance after 3 months of dupilumab 300 mg SC monthly and was successfully tapered off his other immunosuppressive therapies and phototherapy (Table II). At 3-year old we discontinued dupilumab and the patient skin remains clear with application of emollients.

### Case 4

A 5-year-old girl diagnosed with severe medullary aplasia and no past medical history underwent an allogenic HSCT. There was no history of atopy in her family (Table I). On day 32, with tapering of prednisone she presented with diffuse erythemato-squamous AD-like plaques on her face, trunk, and lower limbs. Biopsy was not performed. She showed a partial response to moderate potency topical corticosteroids and TCI. Three months later due to cyclosporin tapering, she had an exacerbation with new plaques on her scalp, trunk, and upper limbs despite compliance with topical treatments. Given her rapid deterioration, dupilumab 300 mg SC each month was started. At 2-months follow-up, a substantial reduction in the number of her skin lesions and her EASI score were noted with pruritus being now absent even with further tapering of her cyclosporine (Table II).

## Discussion

AD-like GvHD is a subtype of GvHD that can be challenging to treat with conventional therapies. Timely diagnosis and treatment of GvHD in pediatric patients is important as a delay in diagnosis can lead to severe and lifelong sequelae for both the skin and various organ systems. The clinical judgment of experienced dermatologists and oncologists without histopathologic confirmation is often the key to the diagnosis. A skin eruption occurring within the first 100 days following HSCT is highly indicative of acute GvHD.^6^ Whereas a skin eruption recurring and persisting beyond the 100-day mark should be evaluated for potential late onset acute GvHD or chronic GvHD based on clinical characteristics.^6^

Skin biopsy seldom leads to a definitive diagnosis of GvHD in children with suspected GvHD lacking specificity.^7^^,^^8^ Experts do not recommend to solely rely on skin biopsy confirmation to guide GvHD treatment since it may not correlate with clinical presentations, particularly in cases where a rash develops a few weeks post-HSCT.^8^ In our cases, the histopathology of both biopsied patients turned out to be AD-like. Two patients developed their rashes a few weeks after their HSCT coinciding with prednisone tapering, while the other 2, who developed it beyond the 100th day, exhibited typical characteristic signs of AD-like GvHD such as eczematous dermatitis with perifollicular accentuation, as well as acral desquamation with eczematous plaques on the trunk and legs (Fig 1). It remains important to emphasize that 2 of our patients displayed a predominantly spongiotic pattern with histology that did not display the typical features of GvHD. It is uncertain whether patients with histology more in line with conventional GvHD features would have responded as optimally.

While the pathogenesis of AD-like GvHD remains poorly understood, research suggests that it may involve an increase in Type 2 inflammation, as evidenced by elevated levels of IgE, Th2 cells, regulatory T cells, and eosinophils in some patients.^9^ An important contribution of Type 2 inflammation in AD-like GvHD may explain the promising response to IL-4 and IL-13 blockade and indicates that different treatment approaches may be required for this subgroup of patients compared to other forms of GvHD. Evidence for involvement of the Type 2 pathway signaling in this GvHD subtype remains to be demonstrated. For example, it is still uncertain whether some patients with AD-like GvHD who do not have eosinophilia and/or increased IgE levels would respond better or less to dupilumab. All our patients had normal eosinophils. Moreover, the findings by Li et al regarding an increase in filaggrin expression in AD-like GvHD patients is intriguing and raises questions about the underlying mechanisms of this condition. The lack of a filaggrin defect in AD-like GvHD patients suggests that the transepidermal water loss in this condition may differ from that seen in atopic dermatitis.^9^

Despite the varied reasons for HSCT in our patients and their refractoriness to diverse combination of conventional GvHD regimen therapies, all 4 achieved near complete clearance, considerable pruritus reduction and improved quality of life under dupilumab. Dupilumab appears to be very promising for the treatment of AD-like GvHD showing greater efficacy than conventional cortisone-sparing treatments such as methotrexate, sirolimus, MMF, cyclosporine, and ruxolitinib. It is intriguing that these agents effective in treating conventional atopic dermatitis or lichenoid/sclerodermoid chronic GvHD, do not seem to achieve the same therapeutic benefits for AD-like GvHD. This phenomenon could also potentially indicate that pruritus may have a primary role in AD-like GvHD, with the eczematous skin changes being a secondary consequence. This hypothesis aligns with well-documented observations from dupilumab treatment, where pruritus often improves before the skin changes become noticeable. It suggests the possibility of a cutaneous neurogenic involvement as in prurigo nodularis.

In addition to the significant decrease in pruritus, and the EASI and Dermatology Life Quality Index scores, dupilumab allowed oncologists to wean off, and in some cases, discontinue immunosuppressive therapies quite rapidly, avoiding long-standing potent immunosuppression, thus reducing the medication burden in these fragile patients. Dupilumab injections are also less time-consuming, do not require serial monitoring blood works and are more convenient than phototherapy and photopheresis for patients with multiple medical appointments or living in remote areas. In multiple clinical trials in AD over the years, dupilumab has shown to be safe, well-tolerated with minimal serious side effects, and has now been approved for children 6-month-old and up. No serious side effects were observed in our patients treated under compassionate care. However, the safety and efficacy of dupilumab in patients with AD-like GvHD, who are immunocompromised due to HSCT, need to be carefully evaluated. To our knowledge, there are no clinical trials employing dupilumab or drug compounds targeting type 2 inflammation to treat any cutaneous form of GvHD.

The limitations of this study are its small sample size, retrospective nature, nonsystematic histopathology studies, and absence of correlation of simultaneous GvHD systemic involvement. However, the main limitation is that we cannot definitively confirm the diagnosis of GvHD in this cohort or distinguish it from the potential alternative diagnosis of atopic or eczematous dermatitis. The diagnosis of AD-like GvHD was a clinical diagnosis in all our patients. Consequently, our findings cannot be extrapolated to suggest that dupilumab may be universally effective for GvHD. We rather suggest the effectiveness of dupilumab in post-HSCT patients with AD-like morphology or and histology.

Dupilumab may represent a promising new therapeutic option for the treatment of patients with AD-like dermatitis post-HSCT. Our case series highlights the successful use of dupilumab in 4 pediatric patients including toddlers with a clinical diagnosis of AD-like GvHD who were refractory to conventional therapies. Further studies are needed to establish the efficacy and safety of dupilumab and other compounds targeting type 2 inflammation in the treatment of AD-like GvHD. Ultimately, clinical trials could lead to a paradigm shift in the treatment of cutaneous AD-like dermatitis post-HSCT.

## Conflicts of interest

Dr Powell, Dr Hatami, and Dr Coulombe received honorarium from Sanofi-Genzyme in advisory boards.

## References

[1] Tanasescu S., Balguerie X., Thomine E. (1999). [Eczema-like cutaneous graft versus host disease treated by UV-B therapy in a 2-year-old child]. Ann Dermatol Venereol.

[2] Wei J., Zhang Y., Xu H., Jin J., Zhang J. (2013). Atopic dermatitis-like presentation of graft-versus-host disease: a novel form of chronic cutaneous graft-versus-host disease. J Am Acad Dermatol.

[3] Link-Rachner C.S., Sockel K., Schuetz C. (2022). Established and emerging treatments of skin GVHD. Front Immunol.

[4] Strong Rodrigues K., Oliveira-Ribeiro C., de Abreu Fiuza Gomes S., Knobler R. (2018). Cutaneous graft-versus-host disease: diagnosis and treatment. Am J Clin Dermatol.

[5] Larijani M., Zarowin D., Wohlschlaeger A., Perman M.J., Treat J.R. (2023). Atopic dermatitis-like graft-versus-host disease treated with dupilumab. Pediatr Dermatol.

[6] Shi C.R., Huang J.T., Nambudiri V.E. (2017). Pediatric cutaneous graft versus host disease: a review. Curr Pediatr Rev.

[7] Haimes H., Morley K.W., Song H., Okhovat J.P., Schmidt B., Huang J.T. (2019). Impact of skin biopsy on the management of acute graft-versus-host disease in a pediatric population. Pediatr Dermatol.

[8] Firoz B.F., Lee S.J., Nghiem P., Qureshi A.A. (2006). Role of skin biopsy to confirm suspected acute graft-vs-host disease: results of decision analysis. Arch Dermatol.

[9] Li K., Mu Z., Wen G., Zhao Y., Cong X., Zhang J. (2020). Increased regulatory T cells and eosinophils characterize atopic dermatitis-like graft-versus-host disease compared with lichen planus-like graft-versus-host disease. J Am Acad Dermatol.

